# 

**Published:** 1883-05

**Authors:** 


### CONTENTS.

### COMMUNICATIONS.

Effects and Causes........ 203
Dentinal Structure, as Related to the "Theory of Heitzmann"...... 212
Exposed Pulp and Treatment........ 219
Address........ 222
Dental Education ........ 233

### SELECTIONS.

Cerebral Dyspepsia........ 246
American Medical Association........ 247
Pasteur's Response to Koch........ 249
Practical Applications of Koch's Discovery........ 250

### EDITORIAL.

Transactions of the Ohio State Dental Society........ 251
Settling Questions ........ 251
The "Microscope"........ 252
A New Disk........ 253
Missouri Dental Journal ........ 254
Examinations........ 254
Illinois State Dental Meeting........ 254

